# Evolvable Smartphone-Based Platforms for Point-of-Care In-Vitro Diagnostics Applications

**DOI:** 10.3390/diagnostics6030033

**Published:** 2016-09-03

**Authors:** François Patou, Fatima AlZahra’a Alatraktchi, Claus Kjægaard, Maria Dimaki, Jan Madsen, Winnie E. Svendsen

**Affiliations:** 1 Department of Micro and Nanotechnology, Technical University of Denmark, 2800 Lyngby, Denmark; faaat@nanotech.dtu.dk (F.A.A.); maria.dimaki@nanotech.dtu.dk (M.D.); winnie.svendsen@nanotech.dtu.dk (W.E.S.); 2 Department of Electrical Engineering, Technical University of Denmark, 2800 Lyngby, Denmark; clkj@dtu.dk; 3 Department of Applied Mathematics and Computer Science, Technical University of Denmark, 2800 Lyngby, Denmark; jama@dtu.dk

**Keywords:** lab-on-chip, smartphone, point-of-care, system evolvability, platform-based design, electrochemistry

## Abstract

The association of smart mobile devices and lab-on-chip technologies offers unprecedented opportunities for the emergence of direct-to-consumer in vitro medical diagnostics applications. Despite their clear transformative potential, obstacles remain to the large-scale disruption and long-lasting success of these systems in the consumer market. For instance, the increasing level of complexity of instrumented lab-on-chip devices, coupled to the sporadic nature of point-of-care testing, threatens the viability of a business model mainly relying on disposable/consumable lab-on-chips. We argued recently that system evolvability, defined as the design characteristic that facilitates more manageable transitions between system generations via the modification of an inherited design, can help remedy these limitations. In this paper, we discuss how platform-based design can constitute a formal entry point to the design and implementation of evolvable smart device/lab-on-chip systems. We present both a hardware/software design framework and the implementation details of a platform prototype enabling at this stage the interfacing of several lab-on-chip variants relying on current- or impedance-based biosensors. Our findings suggest that several change-enabling mechanisms implemented in the higher abstraction software layers of the system can promote evolvability, together with the design of change-absorbing hardware/software interfaces. Our platform architecture is based on a mobile software application programming interface coupled to a modular hardware accessory. It allows the specification of lab-on-chip operation and post-analytic functions at the mobile software layer. We demonstrate its potential by operating a simple lab-on-chip to carry out the detection of dopamine using various electroanalytical methods.

## 1. Introduction

Lab-on-Chip (LoC) technologies are pivotal in the emergence of Point-of-Care (PoC) In Vitro Medical Diagnostics (IVMD) applications. They have been offering increasingly more diverse, integrated and better performing IVMD analytical features. From the early and now common lateral-flow immunoassays, several LoC families have evolved towards complex, instrumented devices relying on actuated flows and advanced biosensing schemes [1]. LoCs have repeatedly demonstrated significant functional capabilities ranging from cell sorting [2], rare cell isolation [3], down to single-cell [4] or single-molecule analyses [5].

Recently, advances in microfluidic Very Large-Scale Integration (mVLSI) [6], droplet [7] and digital-microfluidics [8,9] have opened up new horizons for LoC design, operation and applicability. For instance, by leveraging high component densities [10] and microfluidic logic [11,12,13], mVLSI technology is promoting the emergence of software-programmable LoCs. Instrumented and programmable LoCs have extended the scope of possible on-chip chemical and biological testing at the expense of increased system integration challenges and often of stringent instrumentation and control requirements. As argued by Erickson et al. [14], the costs and complexity of LoC/instrument integration have constituted one of the roadblocks to the generalization of direct-to-consumer PoC, LoC-based systems.

Smart mobile technologies may be transformative in addressing that challenge: the ubiquity, functionality panel and computational power of smart devices make them particularly suitable for coping with the aforementioned instrumentation and control issues. Numerous successful examples of coupled smart device/LoC systems have been described in the literature. A recent review of proof-of-concept systems and applications is available in [15]. Although some smart device/LoC systems do not involve control functions and only make use of the smartphone camera for optical readout purposes, we can anticipate that the increasing level of LoC automation is likely to affect the functional role of smart devices in next generation smart device/LoC systems. A recent example is provided by Li et al. [16], who proposed an Android-based platform allowing the control of a Hardware Accessory (HWA) with embedded solenoid valves and pressure controllers. Several LoC variants can be interfaced to the HWA and execute the appropriate flow actuation scheme required for their correct operation. The sequence of valve actuations and pressure sensor readouts is defined via a programming interface at the mobile Android-software layer.

This example, along with many other recent developments, has partly confirmed the transformative potential of smart mobile technologies for promoting the emergence of home/field-based PoC IVMD testing. Yet another major socio-technical roadblock still hinders the success of direct-to-consumer IVMD systems: their low frequency of use [14]. Unlike glucose monitoring, many IVMD tests only need to be performed sporadically depending on the time scale and clinical significance of the variations exhibited by the biomarkers of interest. As consumer market PoC IVMD systems mainly rely on an instrument/consumable business model, the combined effects of limited system utility, expected low consumable sales volumes and high development costs of systems of increasingly high complexity may jeopardize system adoption and success. We highlighted this limitation in recent publications [17,18] and suggested that de novo system design should be avoided, promoting instead system reuse and incremental system evolution, in order to limit implementation and validation costs, reduce time-to-market, facilitate the integration of new technologies and decrease the risk of early system obsolescence. We argue that system evolvability, defined as “a design characteristic that facilitates more manageable transitions between system generations via the modification of an inherited design and [...] by the ability of an architecture to be inherited and changed across generations [over time]” [19], can assist in that attempt and thus help remedy some of the socio-technical limitations still preventing the generalization of direct-to-consumer IVMD systems. In this paper, we discuss how Platform-Based Design (PBD) constitutes a valid formal entry-point for the design of evolvable systems. We first present the mechanisms by which this may be pursued before illustrating our strategy with a case study: we elaborate on the design and implementation of an evolvable smart device/LoC platform accommodating at this stage high-impedance and electrochemical biosensing.

## 2. Evolvable Systems and Platform-Based Design

Platform-Based Design (PBD) frameworks and methodologies aim at decreasing product development time and the costs of customization [20]. Cost savings are achieved by sharing a common set of components, modules and/or subsystems across various product derivatives. The group of related product variants constitutes the platform product family.

The Google ARA platform, for instance [21], constitutes a particularly interesting example of PBD: it aims at offering extended and user-personalized mobile phone functionality through plug-and-play swappable modules (e.g., camera lens, GPS tracker, etc.). The Google ARA or similar concepts could thus soon support product platforming of LoC-based PoC diagnostic testing, where variants would materialize by modular Hardware Accessories (HWA), themselves interfacing disposable LoCs presenting varying architectures, functions or operation. The differentiation of the LoC modules is to this day still inevitable in order to address specific biological targets and to meet appropriate biosensing performance requirements.

**Figure 1:**
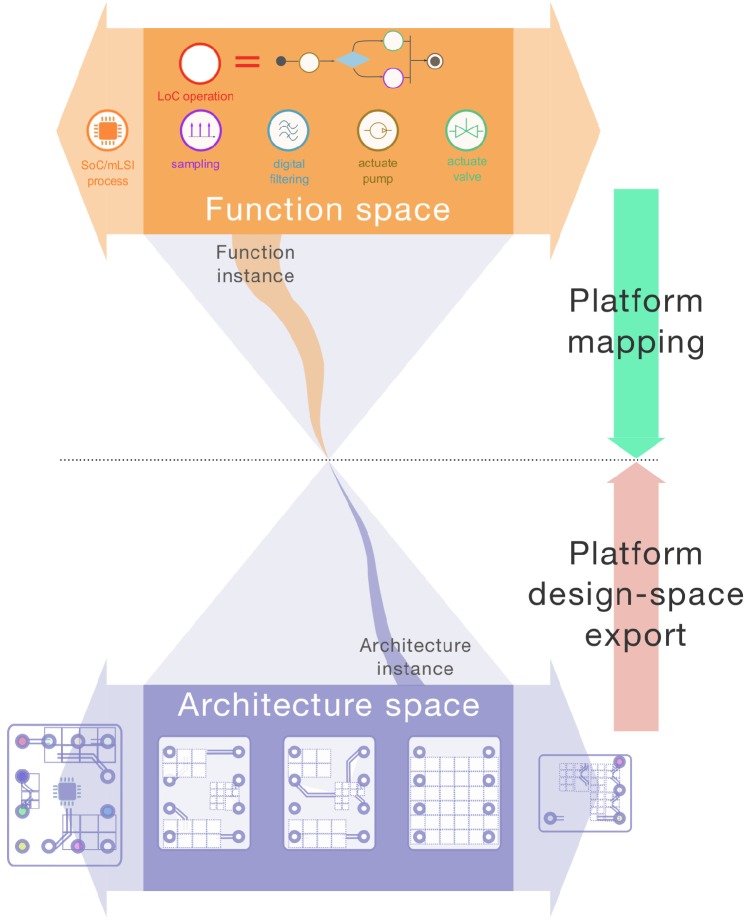
CPS PBD in an uncertain environment. Changing context and requirements may necessitate the expansion of both the function and architectural space. Design principles for favoring platform evolvability are meant to facilitate that expansion at minimum costs and efforts. Adapted from [23].

There is an intrinsic tension in PBD: the will to reuse as many core components as possible between product variants and the wish for the widest panel of variants, which requires singular product characteristics [22]. PBD therefore comprehends all of the challenges of system design, while adding the complexity of platform-specific tradeoffs, such as maximizing commonality while minimizing performance loss, minimizing costs, maximizing variety, etc. [20]. Sangiovanni-Vincentelli famously conceptualized the foundation of PBD for Cyber-Physical Systems (CPS) [23,24]. The PBD methodology for CPS is largely applicable to the design of smart device/LoC system hardware and software. It involves the specification of the platform function space (Figure 1), which comprehends the set of elementary functions that the system must provide in order to fulfil the anticipated use cases. A given function (i.e., function instance) is meant to be allocated to a specific architecture variant. This particular architecture instance should fall within the platform architectural space. This allocation or mapping process is a key feature of CPS platforms. It is often carried out in search of meeting pre-defined optimality criteria (e.g., mapping a function on the architecture that will perform the fastest, etc.).

Although PBD promotes the cost- and time-efficient development of a set of anticipated product variants, it does not intrinsically favor system evolutions when these fall outside of the design space initially considered. The development of LoC variants presenting architectural or functional specificities incompatible with the initial platform hardware and software design specifications may thus require substantial re-engineering costs and efforts. Worse, the standardization of the core hardware and software elements of the platform may actually “limit the innovation and creativity [at the LoC level] by locking the system into specific suppliers and technologies” [25]. Finally, the change of shared hardware/software components in a non-evolvable platform may propagate to several product variants, threatening overall architecture stability or resulting in unbearable costs [26].

PBD can thus represent a powerful strategy to address new needs rapidly and to capitalize on existing developments if these new needs can be satisfied by variants developed within the initial platform function and architecture space. If not, the platform must also be capable of accommodating the new variants in an agile and cost-effective manner: it must be evolvable.

The evolution of CPS platforms can be conceptualized by the expansion of the initial function or architectural space (expansion arrows in Figure 1). Evolvability then translates how smoothly these expansions can occur across platform generations. Among possible strategies to favor smart device/LoC platform evolvability, one may follow Madni’s recommendations [25] to: (1) identify the commonalities shared by the anticipated platform variants, i.e., in functions, structures and operations throughout the the smart device, HWA and LoC sub-systems; (2) locate where in the platform hardware and software architecture change-absorbers and change-enablers need to be implemented; and (3) leverage design principles/systems architectural patterns to implement change-enabling/absorbing mechanisms into the initial platform hardware and software in anticipation of alternative LoC variants.

## 3. Evolvable Platform Early Design Specification

### 3.1. Function Space Definition and Change-Enabling Mechanisms

The specification of the initial function space offered by a CPS platform is determinant in promoting system-level evolvability. Fundamental change-enabling mechanisms should be considered at this early stage. Design principles, such as those suggested by Fricke and Schultz [27] (Table 1), are meant to guide the specification of enduring system architectures. They do not imply specific technologies or implementations.

Non-hierarchical integration: By presenting interfaces at the same hierarchical level, structures or functions can be linked and interact in a more direct manner. LoC, HWA, mobile software and cloud computing functions can, for instance, all be made available to the LoC application designer via interfaces built at the mobile software layer, propagating the specification of LoC variants’ architectures and functionality mapping at a higher level of abstraction.

**Figure 2:**
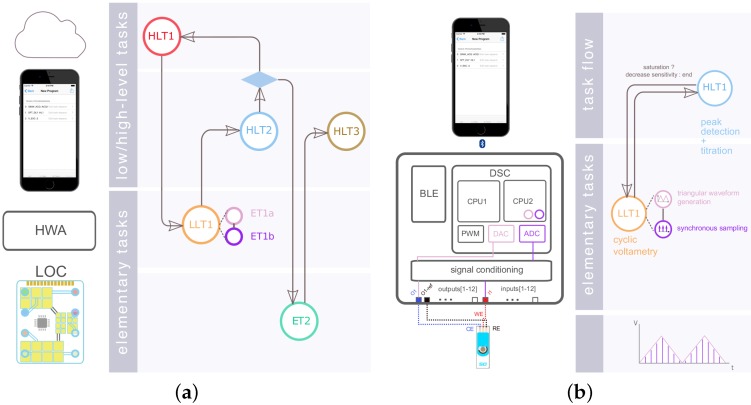
(**a**) Generic illustrative example. The LoC program starts with a high-level task HLT1 carried out by in the cloud followed by a low-level task LLT1 performed by the HWA. A predicate on the execution of HLT2 conditions the next task to be executed: if the program branches to ET2, the program eventually ends by HLT3; otherwise, it loops to the beginning. (**b**) Cyclic voltammetry acquisitions are carried out successively at decreasing sensitivities if current saturation is detected within the potential range where calibration is carried out. Cyclic voltammetry LLT1 is composed of two synchronous elementary tasks: triangular waveform generation and synchronous sampling.

Composability and modularity: Modular system architectures can facilitate the re-use, exchange or adaptation of modules to perform new or more performant functionalities. As detailed in [18], our HWA and software design relies on modular functions, which we distinguish as follows (Figure 2a): Elementary Tasks (ETs) describe physical or computational functions performed at the HWA or LoC level. ETs were defined so as to be composable: they can be composed/associated together while keeping their intrinsic properties, to help realize LoC or HWA physical or computational functions of higher complexity/utility than what each single ET can offer individually. We refer to these compositions as Low-Level Tasks (LLTs) [18,28]. Finally High-Level Tasks (HLTs) are functions that are computational-only and realized in the higher abstraction layers of the system: in mobile software or in the cloud. One of the motivations behind these functional distinctions is the pivotal role of ETs and LLTs in defining overall system operation. HLTs arguably form a vast function space, which may extend from data analytics, to machine learning or cloud computing, whereas ETs and LLTs will be tied to HWA and LoC physical processes, which we expect will considerably constrict the physical function space offered by the platform. The modularity and composability of ETs, LLTs and HLTs should enable the specification of LoC programs, i.e., sequences of tasks required for operating a given LoC variant in an appropriate manner (Figure 2). The platform mapping process then consists of associating each constituent function of an LoC program to the relevant structural component(s).

Independence: Each ET is independent; it alone can provide a useful function without relying on other functions.

Ideality/simplicity: Since ETs will much likely represent the constricting element of the platform function space (they are tied to the physical processes occurring on the LoC or the HWA), they must be carefully defined during initial design. In order to offer the greatest potential for composing LLT functions of enhanced utility, it may be tempting to specify ETs responsible for carrying out very fundamental operations. Let us for instance consider ETs enabling the control of each and every single register of an Analog to Digital Converter (ADC) embedded in the HWA. If they are composable, then the composition of LLTs from these ETs will present the broadest panel of options for controlling the signal acquisition process. Nevertheless, the complexity associated with this functional decomposition may be burdening, since it requires handling low-level embedded software behavior at a higher level of abstraction. The system is highly configurable, but at what cost? On the other hand, if the only ET associated with ADC control triggers an entire sequence of acquisitions with hard-coded settings, then few composition options remain, and it may be difficult to design LLTs offering enhanced utility. The ideality/simplicity principle can guide utility/complexity tradeoffs, suggesting the definition of “only useful/independent functions, which may be interpreted as establishing small, simple units/elements with a minimized number of interfaces (loose coupling among and strong cohesion within modules)”. By defining ideal/simple ETs, we should promote changeability.

### 3.2. Change-Absorbing Interfaces

The design of modular systems requires that the coupling between system modules be minimized while the integrity within modules is maximized. This coupling most often depends on the design of the interfaces associating hardware or software modules between them. Modularity is a powerful concept only if change to a module does not propagate to other modules and if the interfaces between modules remain unaltered. Changing interfaces, especially, is often cumbersome and costly [26]. Lindermann et al. differentiate “local changes”, which are changes confined within a module, from “interface-overlapping changes”, which often become inevitable as the system grows in complexity and connectivity [29]. When properly designed, modular system architectures can facilitate the re-use, exchange or adaptation of modules to perform new or more performant functionalities. The definition of system interfaces is therefore critical in attempting to uncouple system modules and, thus, to limit the propagation of change from one module to the others. Functional or structural module interfaces often behave as change-carriers or multipliers, which means that changing these interfaces may result in cascaded needs for re-engineering other system modules [30]. The two main interfaces of an evolvable smart device/HWA/LoC platform, i.e., the wireless or electrical interface linking the smart device to the HWA and the physical interface coupling the HWA to the LoC, should prevent these cascading effects: they should be designed as change-absorbers.

### 3.3. Architecture Variants and Mapping

**Figure 3:**
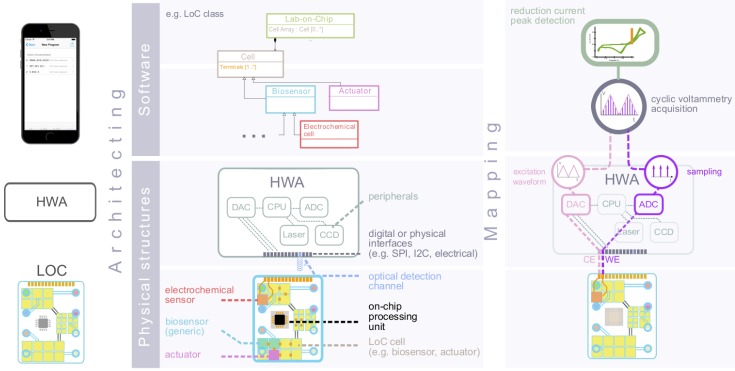
(**a**) Abstraction of the LoC and HWA structural components at the mobile software layer. The physical structures of the LoC and the interfaces enabling their associations are “cyberized” at the mobile software layer [31] in order to enable the definition of LoC variants’ architectures. (**b**) Once both the platform function space and platform architectural space are defined, mechanisms need to be implemented in order to allow the functional allocation of ETs to the relevant structural components of the HWA or LoC: the application mapping.

The definition of the platform’s initial hardware and software function space comes hand-in-hand with the need to adequately abstract the platform architectural space. Both of these processes effectively require to “cyberize the physical” [31] (Figure 3). Specifically, the elementary structural components of a generic LoC, i.e., the LoC cells and the constituents of the interfacing HWA (Figure 3) should be representable at the application layer. These abstracted representations (e.g., biosensors, actuators, embedded processor) should display standardized interfaces to enable their associations, composition, inter-communication, etc. (e.g., the HWA electrical I/O terminals). These, in turn, should make it possible to represent the complete physical architecture of any of the HWA/LoC variants initially considered, as well as alternative variants, unavailable at the initial design time.

The availability of these structural elements and association or composition rules are fundamental to enable the platform design-space export of LoC/HWA variants. This process is key in PBD for CPS: it conditions the automated mapping of platform functions to the target architecture according to pre-defined optimality criteria [24,32]. Formal design-space export methods for optimal mapping were not investigated further within the scope of this work and can be the object of future research.

## 4. Case Study: Evolvable Platform for Current- and Impedance-Based Biosensing

The primary objective of our case study is to illustrate the application of the introduced framework for the design of an evolvable smart device/LoC platform initially enabling LoC application designers to leverage current- or impedance-based biosensing on LoC variants presenting structural, functional or operational specificities. These variants may for instance be differentiated from one another by the number of sensors they embed or by the instrumentation settings for operating these sensors, etc. The second requirement of an evolvable platform should be to facilitate the expansion of both functional and architectural space in anticipation of needed change, such as for the accommodation of a new biosensing technology.

We consider the blank-canvas design of an evolvable smartphone-based IVMD platform relying on the Silicon Nanowire biological Field Effect Transistor (SiNW-bioFET) technology [33]. Largely inspired by the principle of operation of conventional MOS-FETs, SiNW-bioFETs have repeatedly demonstrated their potential for the highly sensitive detection of various biomarkers at ultra-low concentrations [34,35,36,37,38,39,40]. Specific surface chemistries, i.e., biofunctionalization schemes, theoretically allow the adaptation of the sensor to a wide variety of targets and applications. One of the derived requirements for leveraging the SiNW-bioFET technology is to couple it with a sensitive instrumentation in order to be able to recover the concentration of the target analyte from the sub-nanoampere currents flowing through the sensor. The lock-in synchronous detection technique represents a potent instrumentation candidate as it offers both the possibility to recover signals buried in high levels of noise and to reliably quantify signals that may vary in amplitude or frequency over several orders of magnitude [38,39,41,42,43,44,45,46,47]. The technique requires, in our particular case, a sensitive current pre-amplification. This latter requirement constitutes an interesting commonality between SiNW-bioFETs and a variety of other current-based or impedimetric biosensors, including electrochemical biosensors. Electrochemical methods for biological sensing of electro-active compounds have been widely investigated in the field of mobile health in vitro diagnostics testing, mainly on the account of their low cost and relative simplicity [48,49,50,51]. The identification of this commonality served the definition of the initial platform function space.

### 4.1. Change-Enabling Functional and Structural Specification

**Figure 4:**
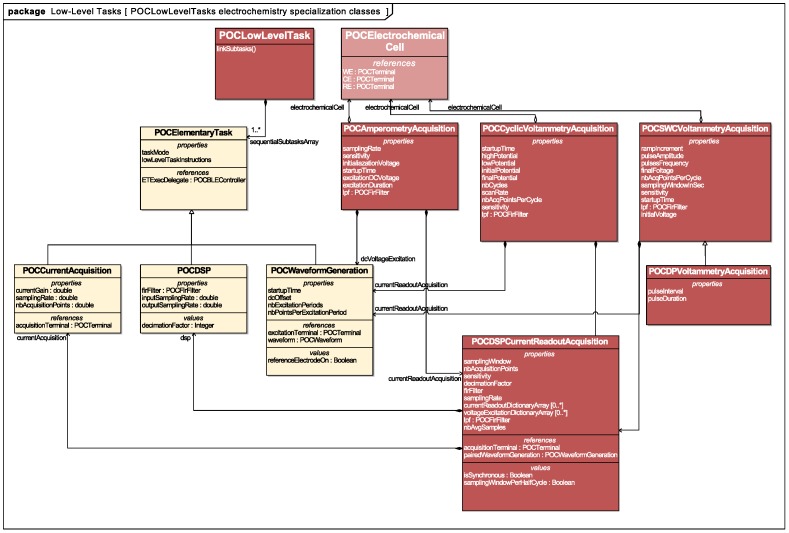
Electrochemical analysis acquisition LLTs’ decomposition.

Functional specification: Rather than specifying a stand-alone lock-in amplification function, we decomposed its functionality guided by the modularity/composability and ideality design principles previously introduced (Table 1 and Table 2). From this functional decomposition, we designed the object-oriented programming classes that would form the initial set of ETs offered by the platform. These classes were made available through an iOS Application Programming Interface (API), enabling the non-hierarchical integration of physical and computational tasks at the mobile software layer. These ETs can be assembled to compose the electroanalytical acquisition functions useful for the operation of electrochemical sensors. A UMLclass diagram representing the relations between objectified ETs and the advanced electroanalytical LLTs is given in Figure 4.

Voltage waveform generation, digital signal acquisition and digital signal processing functions formed the independent, modular and composable functional blocks required for the specification of amperometry, Cyclic-Voltammetry (CV), Square-Wave Cyclic Voltammetry (SWCV), Differential Pulse Voltammetry (DPV), impedance measurements by lock-in amplification and Electrochemical Impedance Spectroscopy (EIS). The design variables for each of these LLTs could be matched to the settings offered by the three ETs (Table 2).

**Figure 5:**
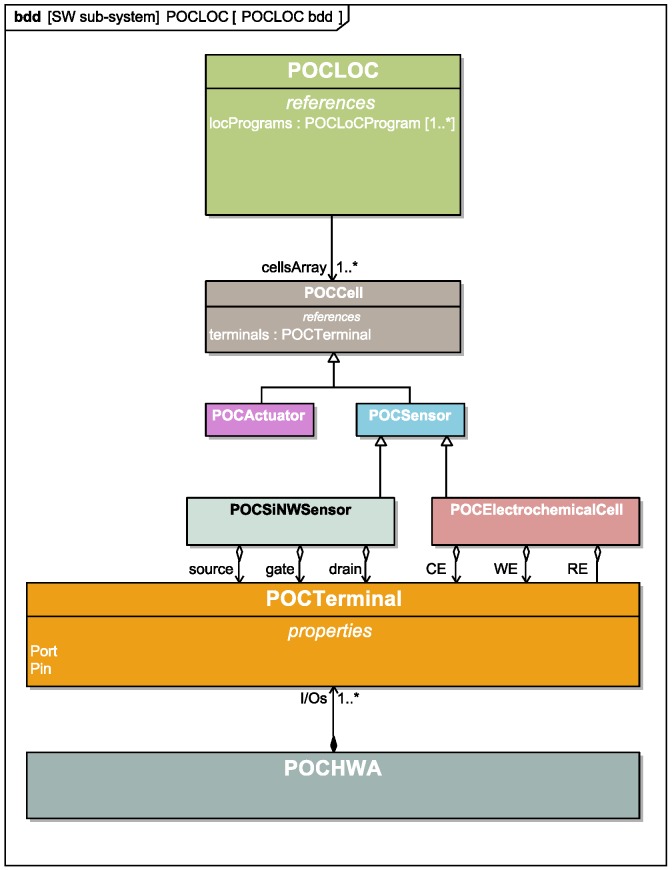
iOS software classes for the main LoC architectural components and their relation to the HWA/LoC interfacing terminals.

Structural specification: As we mentioned earlier, the functionality of a cyber-physical platform is achieved by granting the application designer the possibility to map the available platform functions to a target architecture. As our application software layer is highly abstracted, we leverage object orientation to derive software representations of the physical objects involved in system functionality. The basic abstractions of the LoC, its embedded components and interfaces are presented in Figure 5.

The POCLOC class is compartmentalized into LOCCells. These cells are referenced to in an array structure. POCCell is an abstract class that refers to all of the embedded components on the LoC that play a role in device operation and that may interface to one another via terminals (i.e., POCTerminal class). Terminals may represent physical ports of various natures. Each sensor or actuator embedded in the LoC possesses at least one terminal: the former displays a terminal through which relevant sensing information is retrieved, whereas the latter exhibits a terminal through which actuation can be commanded.

### 4.2. Hardware and Embedded Software Architecture

**Figure 6:**
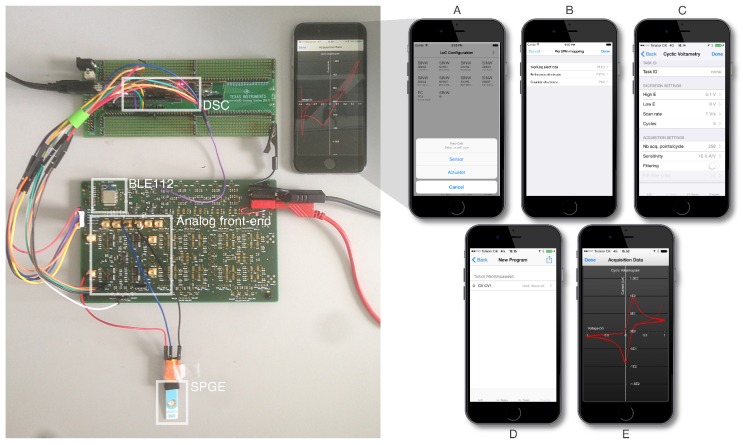
System hardware setup: The HWA materializes at this early development stage by a development board hosting the HWA CPU (TI C2000 TMS320F28377D DSC) interfaced to a break-out PCB embedding the analog signal conditioning circuitry and the BLE module. This PCB is here interfaced to a single SPGE. iOS mobile application storyboard. (**A**–**B**) LoC configuration: define the architecture and configuration of the LoC physically interfaced to the HWA; (**C**) low-level tasks’ definition: select and customize the low-level routines for acquisitions and/or actuation; (**D**) program generation: select either low- or high-level tasks to define the sequence forming the LoC program; (**E**) results: illustration of the cyclic-voltammetry acquisition data.

The performance requirements set by our elementary tasks led us to design an HWA powered at 3.3 V, enabling the single-ended operation of its embedded analog and digital components. It hosts a Bluetooth Low-Energy module (BLE112, Bluegiga Inc., Espoo, Finland) enabling wireless communication with iOS. This module is configured as a master to a dual-core, C2000 Digital Signal Controller (DSC-TMS320F28277D, Texas Instruments Inc., Dallas, TX, USA), so far interfaced via a development breakout board [17] (Figure 6). Communication between the BLE112 module and the DSC is assured using the standard Serial Peripheral Interface (SPI) protocol.

The dual-core architecture of the DSC is leveraged as follows: CPU1 is responsible for the communication with the BLE112 module. As it receives encoded iOS instructions, it translates them into settings for each ET. Upon loading of each ET setting, CPU1 triggers task initialization and execution on CPU2. The DSC second core and its independent hardware accelerator are left responsible for voltage generation functions and digital acquisitions by use of the DSC’s embedded 12-bit DACs or PWM modules, as well as its four embedded 12-bits ADCs in single-ended configuration. Parallelism allows appropriate signal processing routines to be carried out on CPU1 and its own hardware accelerator, while CPU2 keeps running its excitation/acquisition processes, for instance for continuous sensor monitoring applications. Several DC voltages can be output in parallel. The signal processing routines carried out by CPU1 allow digital filtering, decimation and current calibration of the sampled signals. Their output triggers the upstream transmission of processed data to the BLE module that in turn relays them to iOS.

Hardware redundancy, i.e., parallelism, another of Fricke’s design principles for changeability, materializes as follows: twelve buffering operational amplifier stages are connected to the output of both of the DSCs’ DACs and of ten of its PWM modules. These amplifiers were referenced at 1.8 V to offer a theoretical AC output span of up to 1.5 V. Appropriate DAC or PWM waveform generation routines and look-up tables are called depending on the LoC program mapping. Two potentiostatic cells are implemented on the output of both DACs channels as described by others [50], using for each two single-pole single-throw switches to select either a two- or three-electrode electrochemistry configuration.

Twelve current Transimpedance Amplifiers (TIA) (AD8608, Analog Devices Inc., Norwood, MA, USA) with each having four selectable gains ($1\times 10^{4}$ h $3.6\times 10^{6}\;V\cdot A^{-1}$) feed the multiplexed input of the DSC’s ADCs, offering the opportunity to interface transducers presenting currents scaling over several orders of magnitude.

Synchronous acquisitions can be defined so as to couple any of the excitation channels to any of the acquisition channels. These twelve excitation channels and twelve acquisition channels constitute the possible electrical interfaces for mapping current-acquisitions to LoC-embedded sensors.

### 4.3. Change-Absorbing Bluetooth Protocol

The two main interfaces of a smart device/HWA/LoC platform should be carefully designed as change-absorbers. As such, the BLE communication protocol defining the interface between iOS and the embedded firmware is one of the most sensitive throughout the platform. It must have vehicle-encoded information of both LoC-program commands and retrieved data, virtually representing information relevant to the function space, architectural space and mapping between the two.

We designed our BLE protocol as a change absorber, with the objective to prevent the propagation of anticipated changes in the functional or architectural space to other system modules or layers.

The encoding of any information from-to the mobile software layer must be carried onto length-limited opcodes. iOS instructions to the HWA, for instance, are transmitted on the AccessoryConfig20-byte attribute (Table 3).

In order to absorb a potential expansion of the function or architectural space of the platform, the fields carried by AccessoryConfig were represented with a provisional number of extra bits: the Channel mode opcode byte, for instance, is designed to encode the operational mode of a target excitation or acquisition channel in a single byte. Although currently only three modes have been implemented, an entire byte encodes for the Channel mode attribute, making it possible for 255 modes to be represented without having to modify the protocol. If Channel ID points to an acquisition channel, then Channel mode will specify whether the targeted acquisition channel is synchronous to one of the HWA excitation channels and what configuration options are implied. Similarly, the Channel ID attribute is encoded over one byte, enabling the mapping of up to 255 acquisition channels or 255 excitation channels should hardware redundancy be increased without affecting the iOS-DSC firmware interface.

## 5. Operation and Experimental Measurements

We characterized the electrochemical capabilities of our system by interfacing one of its electrochemical measurement channels to Screen-Printed Gold Electrodes (SPGE) (C223AT, DropSens). The SPGEs were cleaned by sonication in ethanol for 10min, then rinsed in distilled water before they were allowed to dry at room temperature. We then proceeded to the electrochemical quantitation of Dopamine (DA) solutions obtained from dopamine hydrochloride (Sigma-Aldrich, Saint-Louis, MO, USA). A stock solution at 0.1 mol · $L^{-1}$ and successive dilutions were prepared to obtain DA concentrations down to $5.9\times 10^{-9}\;\mathrm{mol}\cdot L^{-1}$. We used distilled water (Millipore Milli-Q, Bedford, MA, USA) as a solvent. The dopamine solution was purged with nitrogen before the dilutions were realized.

Amperometry was carried out by setting the electrochemical cell at E=0.6V for 4 s and then applying a potential E=−0.3V for 20 s during which the current decay was measured at 100 samples per second. Quantitation (HLT1 in Figure 2a) was obtained from the integration of the current signal (i.e., charge) over the entire acquisition time. CV was performed using a scan rate of E=100mV · $s^{-1}$ for potentials ranging from E=−0.4V to E=0.4V. HLT1 was designed to detect the reduction current peak between −0.1 and 0.1V. SWCV was configured with the same beginning and end potentials, with increments of $E_{\mathrm{incr}}=5mV$ and pulse amplitudes of 80 mV at a frequency of E=20Hz. DPV was similarly executed from E=0.6V to E=−0.2V with a pulse duration of 0.04 s, an amplitude of E=50mV and a potential step duration of 0.1 s. HLT1 for both SWCV and DPV implemented a differential reduction peak current detection algorithm. The local maximum differential current was probed for [−0.1–0.1] V for SWCV and [0–0.2] V for DPV. Current sensitivities (i.e., reciprocal of the current amplifier gain) were specified programmatically so as to maximize the signal-to-noise ratio for a given DA dilution. Each calibration point was obtained by averaging results from three measurements.

Once the calibration methods were available, we carried out the fully-automated quantitation of two DA solutions with nominal concentrations of $4\times 10^{-3}\;\mathrm{mol}$ · $L^{-1}$ and $40\times 10^{-6}\;\mathrm{mol}$ · $L^{-1}$, respectively. Measurements were performed five times for each solution. Gain/sensitivity was initially set to its maximum value (Gain = $3.6\times 10^{-6}\;V$ · $A^{-1}$). HLT2 replaced HLT1 for this automated quantitation. HLT2 was implemented with a current-saturation detection algorithm in order to determine whether the acquisition should be repeated with a lower input current-gain. Current saturation was probed for within the specified potential range for which we searched for the local maxima/minima. Should saturation be detected, then the LLT was repeated at the directly inferior gain. This scheme was repeated iteratively until no saturation occurred or once the lowest sensitivity was reached ($1\times 10^{-4}\;A$ · $V^{-1}$).

**Figure 7:**
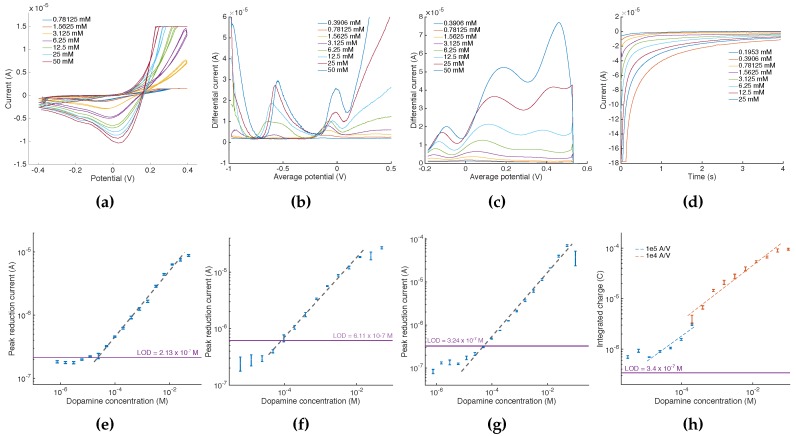
Mobile phone acquisition data. (**a**) Cyclic voltammograms: the calibration point is obtained from retrieving local maxima around E = 0V; (**b**) SWCV acquisitions: the calibration point is obtained from retrieving local maxima around E = 0V; (**c**) DPV acquisitions: the calibration point is obtained from retrieving local maxima for 0 < E < 0.2V; (**d**) amperometry: the calibration point is obtained by integrating the current value for the entire duration of the acquisition. Saturated acquisitions are only discarded when saturation occurs within the potential range of interest. In the specific case of amperometry, acquisitions are discarded if the current is saturated at t = 0 (e.g., acquisition at 25mol · $L^{-1}$ in quadrant d was discarded and repeated with a lower input current sensitivity). (**e**–**h**) Calibration curves for electrochemical scheme respectively illustrated in (a–d).

An illustration of the acquisition data retrieved at the mobile software layer for each method is given in Figure 7. For each acquisition, a calibration point was retrieved from either identifying a local current maxima/minima (i.e., for CV, SWCV and DPV) or by integrating current over the entire acquisition sequence (i.e., for amperometry). From these points, we could derive the calibration curves presented in Figure 7e–h. Regression results are given in Table 4. The calibration curve for amperometry (Figure 7h) was obtained by using a segmented log-log regression, each segment corresponding to the input current sensitivity used for the acquisition. This method allowed us to correct for the total error accumulated from the integration of the current gain and offset errors summed over the acquisition time. For all electrochemical methods, our calibration curves span over several orders of magnitude of DA concentrations with regression coefficients up to 0.996, benefiting from the scalability of the selectable current amplifiers sensitivities.

Finally, the fully-automated quantitation of DA was achieved with accuracies reaching up to 91.8%. By applying a segmented regression (i.e., one for each current sensitivity) also for CV, SWCV and DPV, accuracies could be improved by up to 12.7% for DA at $4\times 10^{-3}\;\mathrm{mol}$ · $L^{-1}$ and up to 19.9% for DA at $40\times 10^{-6}\;\mathrm{mol}$ · $L^{-1}$. Although the achieved LODs do not reach levels documented on other sensing systems (e.g., [52,53,54]), they were obtained from generic commercial electrochemical sensors. This suggests potential for performance optimization, which is out of the scope of this research work.

## 6. Discussion

The framework we propose in this paper aims at promoting system evolvability in smart devices/LoC systems mainly through the implementation of design principles for changeability at the HWA and software level. It thus supplements conventional PBD methodologies for CPS. Assuming the universality of these design principles, we must yet still discuss the implications of their implementation: The non-hierarchical integration of the platform functionality at the mobile software layer provides significant freedom of action as to how to post-process the LoC acquisition data in higher level abstraction layers: in mobile software or potentially in the cloud. This property is valuable in a context where -omics sequencing technologies are increasingly coupled to cloud supercomputing analytics and AI [55,56]. Furthermore, we must not forget that PoC IVMD systems are user-centric systems. The smart device is pivotal in handling user interaction and in articulating the information from chip to cloud. Similarly, the composition of LoC programs intertwining modular low- and high-level tasks offers significant LoC programmability advantages: high-level tasks return values that can be used to modify ensuing LoC program instructions at run time. LoC program conditional branching could thus rely on the evaluation of user-queried or cloud-computing return properties.

This non-hierarchical design yet puts a certain number of constraints on the system requirements, notably on the mobile software/embedded firmware wireless interface. As we mentioned earlier, we strove to design the iOS/embedded firmware protocol as a change absorber to the expansion of the software functional space or to the expansion of the HWA or LoC architectural or functional space. That interface itself must be robust enough to maintain LoC operation even when the smart device (and often its user) is remotely located from the LoC. This, in turn, may imply the wireless transmission of mobile software commands to the HWA via wireless networks and reciprocally the transmission of acquisition data from the HWA to the mobile software layer over these networks. This scenario entails security and privacy concerns, as well as response time considerations should any of the LoC processes be time-critical. We deliberately set these issues aside during our investigation, but we acknowledge that they are of paramount importance.

Our search for modularity enabled us to implement electrochemical capabilities for our platform by the sole functional composition of existing ETs at the mobile software layer and did not require change to any other system components. The composability of ETs is essential for enabling the incorporation of new physical functionality on the platform. It is yet not easily implemented.

Redundancy is probably the most obvious of the design principles favoring evolvability. Although our current platform allows the interfacing of up to 12 current readout channels, many more could have been included with a hardware design inspired from Micro Electrode Array (MEA) systems. One of the arguments against a high level of redundancy/parallelism is the obvious costs of duplicating hardware structures and design controllers capable of running in parallel or in a fast multiplexed manner. In our specific case, duplicating the analog front end current amplifiers alone would at a certain point have required shifting from off-the-shelf components to technologies offering higher levels of integration, such as Application-Specific Integrated Circuits (ASIC), a much costlier design choice. This introduces an important notion in design for evolvability: decisions supporting system evolvability in design must be accompanied both by uncertainty assessment and valuation techniques in order to determine the cost of embedding evolvability at the initial design time, compared to the projected costs associated with the anticipated needed change [57]. Our research work did not address these considerations, rather focusing on where and how could system evolvability be promoted in the initial architecture of smart device/LoC platforms.

The framework we propose in this paper is to our knowledge the first to formally focus on system evolvability as a solution to the socio-technical roadblocks that today still prevent the generalization of LoC technologies in the consumer market. Although we argue this framework can favor the development, adoption and lasting success of next-generation direct-to-consumer PoC systems, we acknowledge that it only partially answers current challenges in IVMD. In particular, it does not substitute the need for cutting edge biosensors and detection methods allowing the recovery of minute quantities of molecules of importance. It also does not account for the integration challenges of these sensing and actuation technologies into small, mass-producible, low-cost LoCs. On that account, our framework can merely inspire LoC designers to work in closer collaboration with instrumentation teams to ensure the overall evolvability both of the platform initially conceptualized and that of the LoC modules themselves. We anticipate that the availability of evolvable PoC instrumentation platforms can encourage the development of new biodetection technologies specifically designed to be compatible with these platforms, thus streamlining IVMD tests development and commercialization.

## 7. Conclusions

We presented in this work a PBD methodology aiming at embedding evolvability in LoC/smart device PoC systems. Our findings should help reinforce the interest for and viability of the PoC IVMD direct-to-consumer business model. We proposed a design methodology for incorporating change-enablers and change-absorbers in smart device/LoC systems hardware and software in search of promoting system evolvability. We demonstrated the applicability of our methodology by implementing a prototype allowing, at this stage, the interfacing of modular, passive LoCs embedding current- or impedance-based biosensors. We finally demonstrated the operation of our platform by carrying out the electrochemical detection of dopamine using various analytical schemes.

## Figures and Tables

**Table 1:** Fricke and Schultz’s design principles for changeability [27].

Ideality/simplicity
Independence
Modularity/encapsulation
Integrability
Autonomy
Scalability
Non-hierarchical integration
Decentralization
Redundancy

**Table 2:** Functional composition of a lock-in amplification acquisition. The acquisition settings, i.e., design variables of the function, can be mapped to the different parameters available through the elementary tasks offered by the platform.

	Initialization Potential	Initialization Duration	Waveform {Sine, Square, Triangle}	Offset Potential	Frequency	Amplitude	Number of Excitation Periods	Ramp Start/Stop Potentials	Duty Cycle	Sampling Rate	Number of Acq.Points	Amplifier Sensitivity	Sample Averaging	Sampling Window	FIRLPF	Decimation	Phase Sensitive Detection
Excitation frequency	—	—	—	—	X	—	—	—	—	—	—	—	—	—	—	—	X
Excitation amplitude	—	—	—	—	—	X	—	—	—	—	—	—	—	—	—	—	X
DC offset potential	—	—	—	X	—	—	—	—	—	—	—	—	—	—	—	—	X
Demodulation frequency	—	—	—	—	—	—	—	—	—	X	X	—	—	—	—	—	X
Sampling order	—	—	—	—	—	—	—	—	—	X	X	—	—	—	—	—	X
Frames per period	—	—	—	—	—	—	—	—	—	X	X	—	—	—	—	—	X
No. of acq. periods	—	—	—	—	—	—	X	—	—	X	X	—	—	—	—	—	X
Sensitivity	—	—	—	—	—	—	—	—	—	—	—	X	—	—	—	—	X
Filtering	—	—	—	—	—	—	—	—	—	—	—	—	—	—	X	—	X
Output sampling rate	—	—	—	—	—	—	—	—	—	—	—	—	—	—	—	X	X
	Voltage Waveform Generation									Current Acquisition					DSP		

**Table 3:** AccessoryConfig attribute value packet structure for synchronous acquisitions specifications. Abbreviations: Channel (Ch.), Decimation factor (Decim.), Acquisition (acq.).

	Byte 0	Byte 1	Byte 2	Byte 3–4		Byte 5–8
Sync.acq.	Ch. ID	Channel mode	Paired exc.channel	No. of acqs.periods		Sampling freq.
	Byte 9 (0:3)	Byte 9 (4:7)–10	Byte 11 (0:3)	Byte 11 (4:7)	Byte 12	Byte 19
	Sampl.order	FPP	Rf gain	FIR sel.	Decim.	Ch.status

**Table 4:** Calibration of the various electroanalytical schemes for dopamine detection. Abbreviations: Single regression (Sing. regr.), Segmented regression (Seg. regr.).

		Calibration Solution			LOD				
		Concentration Range		R^2^	(×10^−6^ mol·L^−1^)	Accuracy (%)			
		min × 10^−6^ mol·L^−1^	max × 10^−6^ mol·L^−1^			@ 4 × 10^−3^ mol·L^−1^		@ 40 × 10^−6^ mol·L^−1^	
						Sing. regr.	Segm. regr.	Sing. regr.	Segm. regr.
CV		97.6	50 × 10^3^	0.982	0.213	86.8	89.3	58.3	78.2
SWCV		48.5	12.5 × 10^3^	0.996	0.611	90	91.8	82.5	87.5
DPV		12.2	50 × 10^3^	0.988	0.324	70	82.7	77.6	86
Amperometry	@ 1 × 10^5^ V·A^−1^	12.2	0.1953 × 10^3^	0.921	0.347	—	—	—	46.7
	@ 1 × 10^4^ V·A^−1^	0.1953	50 × 10^3^	0.977	—	—	86.9	—	—

## Abbreviations

The following abbreviations are used in this manuscript:

ADC: Analog to Digital Converter
AI: Artificial Intelligence
API: Application Programming Interface
ASIC: Application-Specific Integrated Circuit
BLE: Bluetooth Low Energy
CPS: Cyber-Physical Systems
CV: Cyclic Voltammetry
DA: Dopamine
DAC: Digital to Analog Converter
DPV: Differential Pulse Voltammetry
DSC: Digital Signal Controller
ETs: Elementary Tasks
HLTs: High-Level Tasks
HWA: Hardware Accessory
IVMD: In Vitro Medical Diagnostics
LLTs: Low-Level Tasks
LoC: Lab-on-Chip
LOD: Limit Of Detection
MEA: Micro-Electrode Array
mVLSI: microfluidic Very Large-Scale Integration
PBD: platform-based design
PoC: Point-of-Care
PWM: Pulse Width Modulation
SiNW-bioFET: Silicon Nanowire biological Field Effect Transistor
SPGE: Screen-Printed Gold Electrode
SWCV: Square Wave Cyclic Voltammetry
